# RNA Expression Signatures of Intracranial Aneurysm Growth Trajectory Identified in Circulating Whole Blood

**DOI:** 10.3390/jpm13020266

**Published:** 2023-01-31

**Authors:** Kerry E. Poppenberg, Aichi Chien, Briana A. Santo, Ammad A. Baig, Andre Monteiro, Adam A. Dmytriw, Jan-Karl Burkhardt, Maxim Mokin, Kenneth V. Snyder, Adnan H. Siddiqui, Vincent M. Tutino

**Affiliations:** 1 Canon Stroke and Vascular Research Center, University at Buffalo, Buffalo, NY 14203, USA; 2 Department of Neurosurgery, University at Buffalo, Buffalo, NY 14203, USA; 3 Department of Radiology, University of California Los Angeles, Los Angeles, CA 90095, USA; 4 Department of Pathology and Anatomical Sciences, University at Buffalo, Buffalo, NY 14203, USA; 5 Neuroendovascular Program, Massachusetts General Hospital, Harvard Medical School, Boston, MA 02114, USA; 6 Department of Neurosurgery, University of Pennsylvania, Philadelphia, PA 19104, USA; 7 Department of Neurosurgery, University of South Florida, Tampa, FL 33620, USA

**Keywords:** intracranial aneurysm, aneurysm growth and rupture, subarachnoid hemorrhage, RNA-sequencing, transcriptomics, classification

## Abstract

After detection, identifying which intracranial aneurysms (IAs) will rupture is imperative. We hypothesized that RNA expression in circulating blood reflects IA growth rate as a surrogate of instability and rupture risk. To this end, we performed RNA sequencing on 66 blood samples from IA patients, for which we also calculated the predicted aneurysm trajectory (PAT), a metric quantifying an IA’s future growth rate. We dichotomized dataset using the median PAT score into IAs that were either more stable and more likely to grow quickly. The dataset was then randomly divided into training (*n* = 46) and testing cohorts (*n* = 20). In training, differentially expressed protein-coding genes were identified as those with expression (TPM > 0.5) in at least 50% of the samples, a *q*-value < 0.05 (based on modified F-statistics with Benjamini-Hochberg correction), and an absolute fold-change ≥ 1.5. Ingenuity Pathway Analysis was used to construct networks of gene associations and to perform ontology term enrichment analysis. The MATLAB Classification Learner was then employed to assess modeling capability of the differentially expressed genes, using a 5-fold cross validation in training. Finally, the model was applied to the withheld, independent testing cohort (*n* = 20) to assess its predictive ability. In all, we examined transcriptomes of 66 IA patients, of which 33 IAs were “growing” (PAT ≥ 4.6) and 33 were more “stable”. After dividing dataset into training and testing, we identified 39 genes in training as differentially expressed (11 with decreased expression in “growing” and 28 with increased expression). Model genes largely reflected organismal injury and abnormalities and cell to cell signaling and interaction. Preliminary modeling using a subspace discriminant ensemble model achieved a training AUC of 0.85 and a testing AUC of 0.86. In conclusion, transcriptomic expression in circulating blood indeed can distinguish “growing” and “stable” IA cases. The predictive model constructed from these differentially expressed genes could be used to assess IA stability and rupture potential.

## 1. Introduction

Intracranial aneurysms (IAs) are present in 3–6% of the general population, but only approximately 1% of them will ever rupture. Unfortunately, the consequence of IA rupture is subarachnoid hemorrhage, a dangerous condition that is associated with significant mortality and morbidity rates [1]. Thus, it is critical to identify aneurysms most at risk of rupture. Many factors, including patient age and aneurysm characteristics like size and location, are taken into consideration to assess aneurysm risk and determine the best course of action as IA treatment also has risks. Aneurysm growth is a significant risk factor for rupture [2,3,4,5,6,7]; Brinjijki et al. reported growing IAs are more than 30 times more likely to rupture compared to stable IAs [5]. Aneurysm growth currently is assessed by repeat cerebral vascular imaging, typically by computed tomography angiography, magnetic resonance imaging, or digital subtraction angiography [8], which can be expensive and depending on the modality, may carry potential risks.

Over the past few years, we have analyzed gene expression differences in circulating blood and its components between unruptured IA and control cases [9,10,11,12,13,14]. Largely, these transcript panels reflect inflammatory cell activation, cell signaling, and disrupted inflammatory responses. In a study analyzing RNA expression from patients with IA and IA-free controls, we detected 18 genes that were able to identify patients with IA with an accuracy of 85% and an area under the receiver operating characteristic curve (AUC) of 0.91 in an independent testing cohort of samples [9]. More recently, we have developed biomarkers of IA risk as assessed by common clinical metrics, IA size and PHASES (Population, Hypertension, Age, Size of IA, Earlier subarachnoid hemorrhage, and Site of IA) score, in a cohort of IA cases (*n* = 68) [15]. Prediction models based on differentially expressed genes identified between high- and low-risk IAs obtained accuracies >80%. However, these metrics are focused on IA rupture and may not be accurate reflections, as there are reports these metrics underestimate rupture risk [16,17,18,19]. We suspect that IA growth rate may serve as a superior surrogate for IA risk, enabling intervention before rupture.

Here, we propose to identify circulating transcriptome markers to assess IA growth quantified by Predicted Aneurysm Trajectory (PAT), which would allow for more frequent monitoring than imaging alone. PAT is a regression model that uses patient characteristics and aneurysm size to predict growth trajectory of unruptured IAs. Longitudinal data from 520 IAs was the foundation for developing PAT. A transcriptomic analysis also offers the potential to investigate biological mechanisms distinct in growing and stable IAs. Therefore, in this study, we are dichotomizing a dataset of IA whole blood transcriptomes by their PAT scores to identify differentially expressed genes that can be used to build predictive models and to study potentially affected pathways in growing IAs.

## 2. Materials and Methods

### 2.1. Patient Enrollment

The University at Buffalo Human Research Institutional Review Board approved this study (study number 030474433). The study was carried out according to approved protocols. All subjects provided written informed consent. Between December 2013 and September 2018, patients at Gates Vascular Institute (Buffalo, NY, USA) undergoing cerebral digital subtraction angiography were prospectively enrolled for this study. DSA indications included confirmation of IAs identified with noninvasive imaging and follow-up of known IAs. All patients had one or more unruptured IA. For this study, samples were limited to those patients without any potentially confounding cerebrovascular disease or other significant inflammatory conditions.

For each patient, PAT was calculated using the individual’s aneurysm characteristics and demographics for a follow up time of 12 months using the coefficients determined by Chien et al. and presented in a table in their manuscript [20]. The PAT model was built by Chien et al. using multivariate adaptive splines for analysis of longitudinal data in R and is comprised of the following factors: initial IA size, age, sex, cigarette smoking, hypothyroidism, and follow up time in months. In the case of multiple aneurysms, the highest-scoring aneurysm was used. We used median PAT score of the whole dataset to dichotomize IA cases into those with high growth trajectories, i.e., those that are “growing”, and those with low growth trajectories, i.e., those that are more “stable”. 

### 2.2. Whole Blood Collection and RNA Processing 

For this work, 2.5 mL of blood was drawn off the femoral access sheath during DSA and transferred into a PAXgene blood RNA tube (PreAnalytiX, Hombrechtikon, Switzerland). The PAXgene Blood RNA kit was used to extract RNA from blood following manufacturer’s instructions. We removed globin mRNA from samples using magnetic bead capture and the GLOBINclear Kit (Ambion, Austin, TX, USA) according to manufacturer’s instructions. Concentration and purity of isolated RNA was analyzed by absorbance at 260 nm using NanoDrop 2000 (Thermo Scientific, Waltham, MA, USA). Prior to sequencing, RNA concentration was accurately measured by the Agilent 2100 BioAnalyzer RNA 6000 Pico Chip (Agilent, Las Vegas, NV, USA), while purity was precisely assessed by the Quant-iT RiboGreen Assay (Invitrogen, Carlsbad, CA, USA). Samples of sufficient quality (260/280 ratio ~2, RNA integrity number ≥ 6.0) were selected for RNA-sequencing (RNAseq). 

### 2.3. RNA Sequencing Analysis

RNA libraries were prepared with the Illumina TruSeq stranded total RNA gold kit (Illumina, San Diego, CA, USA). RNA-sequencing was performed on the Illumina NovaSeq6000 or the Illumina HiSeq2500 system in two batches. Samples were demultiplexed with Bcl2Fastq. Per-cycle basecall files generated by the NovaSeq6000 were converted to pre-read FASTQ files using bclfastq version 2.20.0.422 under default parameters. FastQC v.0.11.5 was used to assess sequencing quality, while FastQ Screen v.0.11.1 was used to detect potential contamination. Genomic alignments were performed using HISAT2 v.2.1.0 under default parameters. NCBI reference GRCh38 was used for the reference genome and gene annotation set. Sequence alignments were compressed and sorted into binary alignment map files using samtools v.1.3. Mapped reads for genomic features were counted using Subread featureCounts v.1.6.2 using the parameters -s 2 -g gene_id -t exon -Q 60; the annotation file specified with -a was the NCBI GRCh38 reference from Illumina iGenomes. ComBat-seq was used in R to correct raw counts of protein-coding genes with a sum > 0 across all samples for any bias introduced by sequencing in different batches. 

Corrected counts were then normalized as transcripts per million (TPM) using the convertCounts function in R. We then further investigated the contribution of each 6 major cell types in these whole blood samples: B cells, CD8+ T cells, CD4+ T cells, Natural Killer cells, Monocytes, and Macrophages. Using all genes with an approved HGNC symbol and the LM6 signature, we ran CIBERSORT digital cytometry separately on “stable” and “growing” aneurysm samples. 

### 2.4. Differential Expression Analysis 

We randomly divided our dataset into training (*n* = 46) and testing (*n* = 20) cohorts, withholding the testing cohort from training to facilitate independent testing. After limiting training dataset to genes expressed in at least 50% of the samples (TPM > 0.5), we identified differentially expressed genes (DEGs) between IAs with high and low PAT scores. Differential gene expression analysis was performed on TPM data using modified F statistics to assess variation in the mean on a gene-by-gene basis. Multiple hypothesis testing correction was performed using Benjamini-Hochberg false discovery rate (FDR) correction. Genes with an FDR-corrected *p*-value (*q*-value) < 0.05 and an absolute fold-change in mean expression ≥ 1.5 were considered significantly differentially expressed. To visually assess how differential expression separated “growing” and “stable” IAs, we performed principal component analysis using prcomp in R and one minus Pearson correlation (log normalized) hierarchical clustering using the Broad Institute’s Morpheus application.

### 2.5. Bioinformatics Analysis

To study the biological mechanisms of the PAT-associated genes, we performed bioinformatics analyses on the differentially expressed genes using Ingenuity Pathway Analysis (IPA). We queried the disease and biological function terms, considering those with a Benjamini-Hochberg *p*-value < 0.05 and at least 3 input genes. We also used IPA to create networks of potential gene interactions by mapping each gene’s identifier to its corresponding gene object in the Ingenuity Knowledge Base and overlaying them onto identified molecular networks in the database. Gene networks were algorithmically generated based on their “connectivity” derived from known interactions between the products of these genes. We considered networks with a *p*-score > 21 to be significant. 

### 2.6. Classification Model Generation

We used MATLAB’s Classification Learner App to investigate potential of differentially expressed genes to discriminate between “growing” and more “stable” IAs. TPM expression data of the DEGs identified in the training cohort was input into MATLAB for both training and testing sample sets. Here, we implemented a subspace discriminant ensemble model with 30 learners with a 5-fold cross-validation scheme during model training. The model was evaluated in both the training and testing datasets by calculation of the area under the receiver operating characteristic (ROC) curve (AUC), as well as computation of accuracy, sensitivity, and specificity, which were summarized in a confusion matrix. 

We also used Seurat [21] and UMAP [22] to perform dimensionality reduction and visualize how all the samples (based on expression of the DEGs) distribute in terms of PAT score, as an unbiased way of measuring dose-response to our outcome variable. Here, we performed parametrized, density-based clustering of the samples, and PAT score was withheld from this unsupervised analysis. A 2D projection of the data, based on the top 2 UMAP components (UMAP 1 and UMAP 2) was visualized, and the holdout feature (PAT score) was superimposed as a scaled colormap, with red indicating higher risk and blue indicating lower risk. In an effort to identify genes that may be most strongly associated with IA risk, we also compared the PAT-associated genes identified here with those we found for clinical metrics of IA risk: size and PHASES (Population, Hypertension, Age, Size, Earlier subarachnoid hemorrhage, and Site score) [15].

## 3. Results

### 3.1. Study Population 

We analyzed whole blood samples from 93 individuals with unruptured IAs receiving DSA, of which 27 were excluded due to potentially confounding cerebrovascular or other inflammatory conditions. Demographic and comorbidity information for the 66 samples analyzed in this study are presented in Table 1. Based on a follow-up of 12 months, PAT scores ranged from 1.55 to 19.80. Using the median to dichotomize into stable and growing IAs resulted in 33 samples in each group. The 66 RNA samples were of high quality with an average 260/280 of 1.98 and an average RIN of 8.47. Sequencing was successful with an average alignment rate of 96.64%. RNA quality and sequencing metrics are presented in Supplemental Table S1.

Furthermore, we used CIBERSORT to investigate the cellular profile of our whole blood samples. In both “growing” and “stable” groups, neutrophils were the greatest contributors with 53% and 54% respectively, followed by CD8+ T cells, CD4+ T cells, monocytes, natural killer cells, and B cells which only represented about 5% of the population, as shown in Supplemental Figure S1. There was no significant difference between IAs that had high PAT vs. low PAT for any of the cell types examined.

### 3.2. Differentially Expressed Genes between High Growth and Low Growth Cases

**Figure 1:**
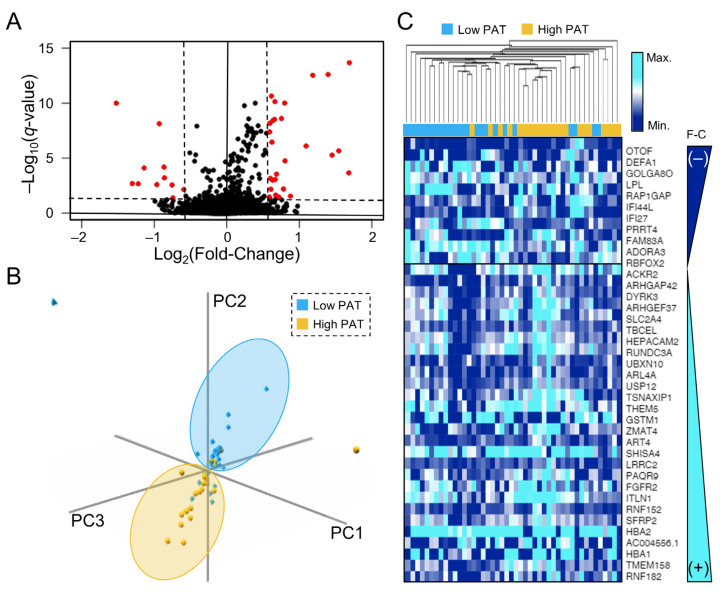
Differential Gene Expression Analysis. (**A**) The volcano plot demonstrates differential expression between “growing” and “stable” IAs as assessed by PAT score. Differentially expressed genes in red have a *q*-value < 0.05 and an absolute fold-change ≥ 1.5. (**B**) Three-dimensional principal component analysis demonstrates separation between low-risk (more “stable” IAs) and high-risk (high PAT, “growing” IAs) cases based on expression of differentially expressed genes in training dataset. (**C**) The heatmap demonstrates separation between low-risk and high-risk IAs based on expression of DEGs in training. Hierarchical clustering groups most low-risk samples on the left side, while high-risk are on the right. (Abbreviations: PAT = predicted aneurysm trajectory, DEG = differentially expressed gene, PC = principal component, F-C = fold-change, max. = maximum, min. = minimum).

RNA-sequencing data encompassed 66,023 transcripts, of which 47,088 had detectable expression (sum > 0 across all samples). Furthermore, 18,729 mapped to protein-coding genes, of which 13,518 had expression (TPM > 0.5) in at least 50% of samples. Our analyses were performed in this refined dataset. Expression differences between growing and stable IAs are illustrated in the volcano plot in Figure 1A. In training cohort, we identified 39 differentially expressed genes (absolute fold-change > 1.5, *q*-value < 0.05) between high PAT and low PAT IA cases (see Table 2). Eleven genes had reduced expression in IAs with high growth trajectories, while 28 had increased expression. As seen in the PCAs in Figure 1B, this panel of genes is able to separate the two classes in the training cohort; the division between the groups was also evident in the testing dataset (see Supplemental Figure S2). Additional unsupervised analysis was performed by hierarchal clustering, that also grouped cases of low and high PAT together (see the heatmap in Figure 1C where the genes with decreased expression are at the top and the genes with increased expression are towards the bottom).

### 3.3. Bioinformatics Analysis

**Figure 2:**
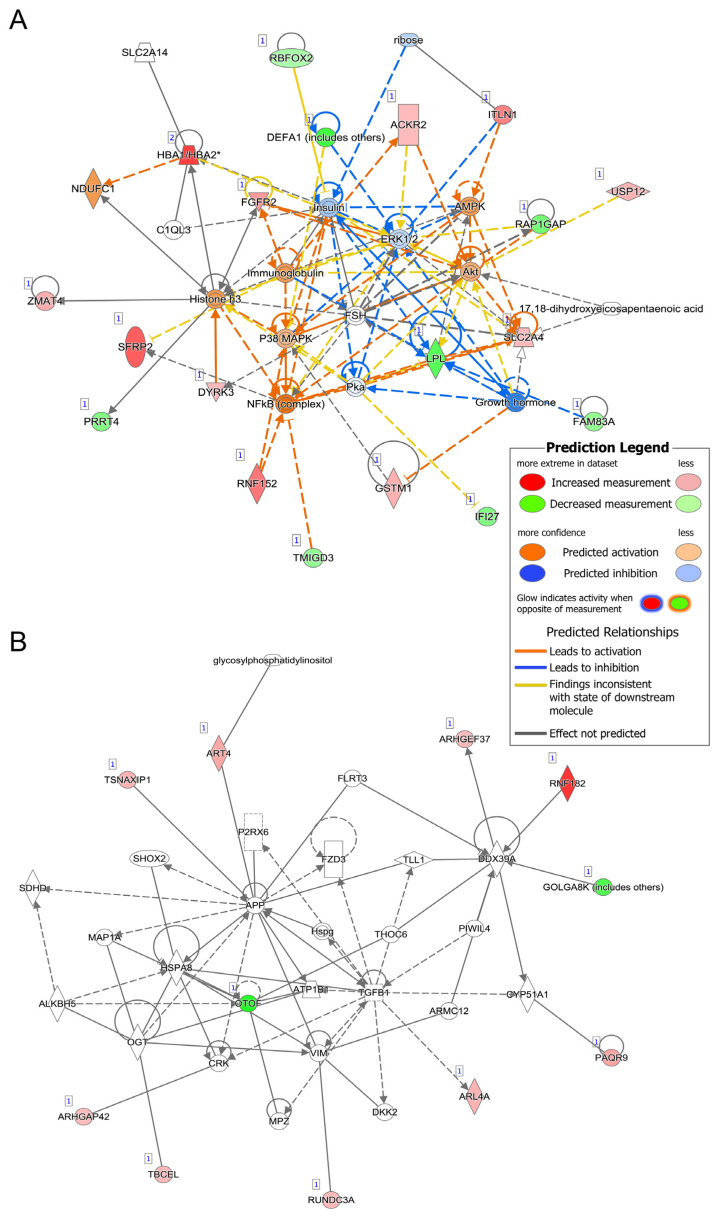
Ingenuity Pathway Analysis Networks. Networks derived from Ingenuity Pathway Analysis based on differentially expressed genes associated with PAT score. For the DEGs (indicated by [1]), red indicates increased expression in high-risk aneurysm cases, while green denotes decreased expression; the color intensity reflects fold-change. Direct relationships are represented by solid lines, while indirect ones are represented by dashed lines. (**A**) The first significant network had a *p*-score of 49 and was related to organismal injury and abnormalities. (**B**) The other significant network (*p*-score = 25) was associated with cardiovascular system development and function and cellular movement. (Abbreviations: PAT = predicted aneurysm trajectory, DEG = differentially expressed gene).

Network analysis was performed to examine potential interactions between the gene set and terms identified in the Ingenuity Knowledge Base. As shown in Figure 2A,B, there were 2 significant networks of genes (*p*-score > 21) associated with the DEGs. The first network (Figure 2A, *p*-score = 49) was densely connected and was associated with “dermatological diseases and conditions”, “nutritional disease”, and “organismal injury and abnormalities”, with inflammatory signaling molecule nodes, including NFKB, ERK1/2, and immunoglobulin. The second network (Figure 2B, *p*-score of 25) was associated with “cardiovascular system development and function”, “cellular movement”, and “molecular transport” processes, and had nodes of interaction around TGFB1 and APP. Networks are summarized in Supplemental Table S2. Genes differentially expressed between the two groups were significantly enriched for several disease and biological function terms, including those related to cancer and organismal injury, cell to cell signaling and interaction, and tissue development. Supplemental Table S3 reports all significant disease and biological function terms.

### 3.4. A Classification Model of IA Growth Trajectory

**Figure 3:**
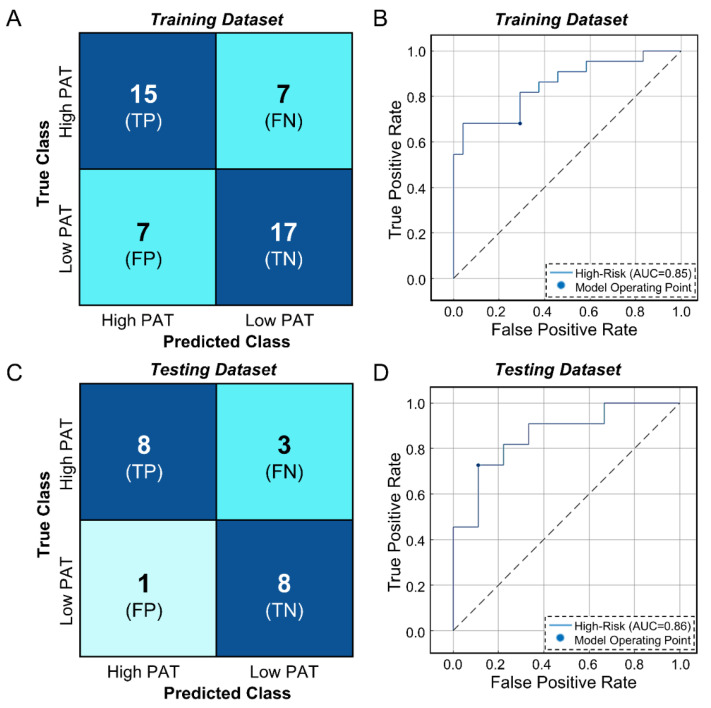
Subspace Discriminant Ensemble Model Performance. The model built using PAT-associated genes was evaluated in separate training (by 5-fold cross-validation) and testing datasets. The model performed well in the training dataset (*n* = 46) as seen in the confusion matrix (**A**) and ROC curve (**B**), which obtained an AUC of 0.85. The model also achieved high performance in testing, as reflected by the confusion matrix (**C**) and ROC curve (**D**), which had an AUC of 0.86. The dotted line in the ROC curves represents random chance. (Abbreviations: PAT = predicted aneurysm trajectory, ROC = receiver operator characteristic curve, AUC = area under the curve, TP = true positive, TN = true negative, FP = false positive, FN = false negative).

The classification model trained using the DEGs performed well in both training (assessed by 5-fold cross-validation) and independent testing. In the training dataset, the model had an accuracy = 69.6%, a sensitivity = 0.68, a specificity = 0.71, and an AUC = 0.85 (Figure 3A,B). In the testing dataset the model was also successful with an accuracy = 80%, a sensitivity = 0.71, a specificity = 0.89, and an AUC = 0.86 (Figure 3C,D). This model shows the potential of this gene panel to assess IA growth via PAT score. As default settings were used in MATLAB, further work could be done to optimize parameters and augment model performance.

**Figure 4:**
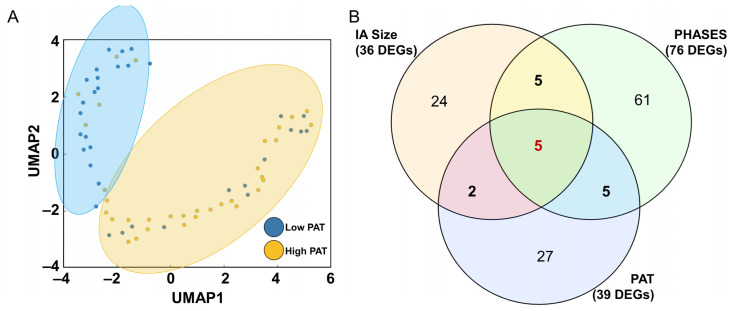
Additional Exploration of PAT-Associated Genes. (**A**) UMAP visualization of differentially expressed genes with PAT score superimposed as a scaled colormap, which shows separation between low (more “stable”) and high (“growing”) PAT samples. (**B**) The Venn diagram depicting which DEGs were also identified as differentially expressed in whole blood transcriptomes between high and low risk IAs, when risk is defined by either IA size, PHASES score, or the PAT. Five of the DEGs were common across all comparisons. (Abbreviations: PAT = predicted aneurysm trajectory, UMAP = uniform manifold approximation and projection, IA = intracranial aneurysm, PHASES = Population, Hypertension, Age, Size, Earlier subarachnoid hemorrhage, site).

We further performed dimensionality reduction to visualize how all samples were distributed (based on DEGs) according to their PAT score. The collective expression of the DEGs was able to separate patients into “growing” (high PAT score) and more “stable” (low PAT score) groups as seen in Figure 4A. Additionally, we compared the differentially expressed genes associated with PAT score to the genes from our previous work which focused on clinical metrics of IA risk, size and PHASES. As seen in the Venn diagram in Figure 4B, there are 12 genes in common between PAT and size/PHASES panels, 5 of which were differentially expressed in all 3 metrics (*DEFA1*, *HBA1*, *HBA2*, OTOF, SFRP2). Furthermore, 10 of the 12 genes have the same fold-change direction across these different datasets, i.e., *DEFA1* is decreased in high-risk aneurysms whether assessed by PAT, IA size, or PHASES score.

## 4. Discussion

After an IA is detected, neurointerventionalists must consider the risks of rupture and treatment complications as they decide the best approach for each patient. Towards a personalized medicine approach, we have been exploring circulating biomarkers of rupture risk, which could complement current diagnostic imaging paradigms by offering neurointerventionalists a non-invasive, biologic method to assess IAs. While there have been several efforts in developing such biomarkers over the past couple of decades, the endeavor has persistently faced the challenge of a lack of longitudinal data within which biomarkers of risky IAs could be developed. Typically, current markers have been developed using cross-sectional data, which typically only contain “snapshots” of unruptured IAs and their morphology at a single time point. In our previous work we identified gene panels and built computational model to assess IA risk through surrogate variables, namely IA size (established in the ISUIA study [23]) and the PHASES score [15]. Yet, these models are only predicting the metric itself and may not accurately reflect the potential for IA rupture in the future. Moreover, these risk metrics were developed in cohorts where the most rupture-prone, dangerous IAs were likely treated and removed from the analysis, introducing selection bias.

To overcome these challenges, we quantified aneurysm growth potential (instability) by calculating the PAT [20] for each case in this study. Trained and validated in a dataset of 520 longitudinally followed IA cases, PAT calculates the rate at which a given IA will grow. This model accounts for several aneurysm and patient specific factors, which were evaluated across a stepwise procedure to remove non-significant and collinear factors. In all, smoking and hypothyroidism had significant effects on the growth trajectories of larger IAs (≥7 mm). Initial aneurysm size was also significantly related to growth, as larger IAs tended to grow significantly faster than small IAs (<3 mm) [20]. Moreover, IAs in males showed faster growth rates than IAs in females. All these factors were considered in the PAT, which Chien et al. [20] further showed was markedly greater in ruptured IAs as compared to unruptured IAs. Thus, in this study, we calculated PAT for all aneurysms in our dataset and performed differential expression analysis, identifying 39 DEGs to be associated with PAT score in the training cohort. Using these genes, we trained and tested a prediction model, which achieved > 80% average testing accuracy and an AUC = 0.86 in the independent testing dataset.

We suspect that the identified DEGs were able to robustly classify higher-risk, high PAT IAs because they captured critical pathways and processes connected to IA progression and rupture. Bioinformatic analyses show that differentially expressed genes were related to two general biologic phenomena, namely organismal injury and abnormalities (mostly with respect to cancer and cardiovascular disease) and cell-to-cell signaling. Another inflammatory disease that was associated with organismal injury and abnormalities in our data was systemic lupus erythematous (SLE). As found in our previous analysis of a PHASES-associated gene panel [15], SLE was also a significant disease function, likely reflecting that individuals with the disease are more prone to rapid IA growth and rupture [24,25]. Many other terms in this category reference the gene *IFI27*, an interferon-inducible protein affiliated with innate immune system, interferon gamma signaling, and apoptotic signaling. Indeed, apoptosis of mural and intimal cells is a critical contributor to medial thinning and destructive remodeling in IA pathogenesis [26]. *IFI27* has also been reported as differentially expressed between chronic ruptured aneurysm and control blood samples [27], further suggesting its importance in IA progression to rupture. Another noteworthy gene reflected in many of the organismal injury and abnormalities terms was *ARHGAP42*, a rho GTPase-activating protein that is enriched in vascular smooth muscle cells. During IA natural history vascular smooth muscle cells (vSMCs) undergo phenotypic modulation to a pro-inflammatory state, which includes increased expression of matrix metalloproteinases and subsequent destructive remodeling [28,29,30]. 

Other genes related to organismal injury and abnormalities included several inflammatory molecules. Certainly, cytokines and chemokines also aid in the inflammatory response and cell recruitment in IA natural history [31]. *ACKR2*, an atypical chemokine receptor that binds inflammatory cytokines, was identified as differentially expressed between “stable” and “growing” IAs. *ACKR* genes are important regulators of chemokine signaling and are predominantly expressed by vascular endothelial cells and some leukocytes [32]. Additionally, our analysis identified *SFRP2* (which was called in many of the organismal injury and abnormalities IPA terms) as an overexpressed DEG in high growth IAs. *SFRP* genes act as modulators of Wnt signaling and have been found to be overexpressed in certain types of cancers. In IA, *SFRP2* was among the top 5 genes overexpressed in IA tissue compared to controls in a study by Kleinloog et al. [33,34] and has been found to be increased in IAs with high aspect ratios [35], an anatomical characteristic that is higher in ruptured IAs [36].

In addition to organismal injury and abnormalities, many other model genes were related to cell to cell signaling and interaction. The genes most associated with these signaling terms were *FGFR2*, *RAP1GAP*, *ITLN1*, and *LPL*. Most notably, *FGFR2* has known roles in angiogenesis, wound healing, and cell migration. In aneurysm pathogenesis, inflammatory cells migrate to the aneurysmal lesion, perpetuating the inflammatory response and destructive remodeling, including matrix degradation and internal elastic lamina disruption [31]. The DEG *ITLN1* is associated with the innate immune system and defensins, which are predominantly found in neutrophils [37]. *ITLN1* has been reported to be differentially expressed in aneurysm tissue compared to control superficial temporal artery tissue, indicating it may be related to IA-lesion specific signaling at the IA site [38].

Interestingly, *DEFA1*, or defensin alpha 1, was also found to be differentially expressed between IAs with high PAT and low PAT. DEFA1 protein is abundant in the microbicidal granules of neutrophils and has been known to play a role in phagocyte-mediated host defense. This gene, which was found to have lower expression in the high growth rate IAs in our analysis, has also been found to be decreased in ruptured IAs at a proteomic level [39]. In our previous work analyzing profiles of circulating neutrophils and PBMCs from patients with IA (compared to IA-free controls), we also found that *DEFA1* was significantly differentially expressed [10,40]. Through these multiple studies studying various analytes, it appears that *DEFA1* plays a critical role in IA progression towards rupture. However, longitudinal studies and mechanistic investigations in animal models are needed to better understand the role of *DEFA1* in IA.

Comparison of the DEGs associated with PAT and those that have been identified between high- and low-risk IAs, as assessed by IA size or PHASES score [15], showed several genes common among all comparisons. This included *DEFA1*, *HBA1*, *HBA2*, *OTOF*, and *SFRP2*. With the exception of *OTOF*, all genes had the same fold-change in direction across the three analyses. These 4 genes (*DEFA1*, *HBA1*, *HBA2*, *SFRP2*) may have the best prognostic potential, as they may reflect processes associated with IA growth and rupture potential that each ground truth metric (PAT, IA size, and PHASES) assesses. As detailed above, *DEFA1* and *SFRP2* may indicate a role for dysregulated inflammatory processes and Wnt signaling in IA, respectively. *HBA1* and *HBA2* both encode alpha subunits of hemoglobin and have also been found to be increased in abdominal aortic aneurysms [41,42]. Aside from its oxygen carrying function, hemoglobin has been found to be a regulator of iron metabolism and an antioxidant in different tissue types [43,44], which may play a role in the inflammation associated with IA. Similarly, other genes that may have prognostic potential are *IFI27* and *LPL*—a.k.a. Phospholipase A1 (common among DEGs between low and high PAT and large and small IAs), as well as *FAM83A*, *GSTM1*, *RNF152*, *RNF182*, *RUNDC3A* (common between low and high PAT and low and high PHASES comparisons). Although, these genes may have been identified because of shared features in the calculation of the risk assessment metrics, for example patient age and IA size. Nonetheless, because these DEGs may reflect biological mechanisms associated with rapidly growing IAs, further longitudinal studies are needed to establish this mechanistic relationship. 

This study has several limitations. First, all samples were collected from a single center. We are currently conducting multi-center studies to help eliminate potential selection bias. Second, the sample size of our study was small. In the future, studies in larger, multi-center datasets will be required to assess true biomarker capability. Third, the PAT score is a surrogate of IA growth and rupture risk. More exact biomarkers will be needed to better predict growing from stable IAs. Large, longitudinal studies will be required to better assess the diagnostic accuracy of this growth surrogate biomarker. With longitudinal samples, we could assess true growth rates rather than those predicted by PAT score and analyze potential gene expression correlation with growth on a continuous scale. Furthermore, with a larger sample size, we can account for other risk-associated parameters that may contribute to differential expression identified here, such as IA location as those in posterior circulation tend to be larger and have a greater rupture risk [45]. Lastly, the DEGs identified here may overlap with those identified in other vascular diseases, which could limit the specificity of the biomarker. For example, several (5/39) of the significant genes (including *LPL* [46], *IFI44L* [47], *ACKR2* [47], *HBA1* [41], and *HBA2* [42]) have also been found to be differently expressed in abdominal aortic aneurysms. 

## 5. Conclusions

In conclusion, transcriptomic signatures derived from circulating whole blood of individuals with intracranial aneurysms can be used to identify and predict those with IAs with higher growth potential, as assessed by the PAT score. A subspace discriminant model built using the 39 genes identified as differentially expressed between low and high PAT IAs achieved AUCs ≥ 0.85 in both training and testing. Moreover, the genes as a whole reflect broad processes, such as organismal injury and abnormalities. On a feature level, multiple genes, namely *DEFA1* and *SFRP2*, may be key to the prognostic power of the model, as they are tied more closely to biologic processes involved in IA pathogenesis. After validation in larger datasets, this model could be used to assess IA growth potential prior to cerebral imaging or permit more regular monitoring for those in which treatment is not deemed appropriate. We hope that such a blood-based prognostic marker could facilitate superior IA management, thereby reducing the number of IAs that rupture. 

## Figures and Tables

**Table 1:** Patient and Aneurysm Characteristics *.

Characteristic	Training Dataset (*n* = 46)	Testing Dataset (*n* = 20)	*p*-Value
Age (average years ± s.d.)	57.2 ± 12.2	58.9 ± 13.5	0.62
Female (*n*/*n*_total_)	36/46 (78.3%)	13/20 (65.0%)	0.26
Smoking (*n*/*n*_total_)	12/46 (26.1%)	3/20 (15.0%)	0.32
Hypertension (*n*/*n*_total_)	19/46 (41.3%)	11/20 (55.0%)	0.30
Family history of IA (*n*/*n*_total_)	8/46 (17.4%)	0/20 (0.0%)	0.05
Patients with multiple IAs (*n*/*n*_total_)	8/46 (17.4%)	5/20 (25.0%)	0.48
IA location (*n*/*n*_total_)			
ACA/ACom	7/59 (11.9%)	2/26 (7.7%)	0.56
BA/BT	5/59 (8.5%)	2/26 (7.7%)	0.90
ICA	27/59 (45.8%)	15/26 (57.7%)	0.31
MCA	10/59 (16.9%)	2/26 (7.7%)	0.26
PCA/PCom	10/59 (16.9%)	5/26 (19.2%)	0.80

* Clinical factors were retrieved from patients’ medical records. Excluding age and IA size, datapoints were quantified as binary factors. There was no statistical significance between the two datasets across all patient characteristics (*p* < 0.05, χ-squared test except for “age”, which used Student’s t-test). Abbreviations: IA = intracranial aneurysm, *n* = number, s.d. = standard deviation, ACA = anterior cerebral artery, Acom = anterior communicating artery, BA = basilar artery, BT = basilar terminus, ICA = internal carotid artery, MCA = middle cerebral artery, PCA = posterior cerebral artery, PCom = posterior communicating artery.

**Table 2:** Differentially Expressed Genes Associated with IA Growth Trajectory *.

Name	Gene ID	Log_2_(F-C)	*q*-Value
ENSG00000115155.16	*OTOF*	−1.52	1.00 × 10^−10^
ENSG00000206047.2	*DEFA1*	−1.30	2.06 × 10^−3^
ENSG00000206127.10	*GOLGA8O*	−1.22	2.19 × 10^−3^
ENSG00000175445.14	*LPL*	−1.14	7.92 × 10^−5^
ENSG00000076864.19	*RAP1GAP*	−0.96	2.59 × 10^−3^
ENSG00000137959.15	*IFI44L*	−0.93	7.39 × 10^−9^
ENSG00000165949.12	*IFI27*	−0.87	6.56 × 10^−5^
ENSG00000224940.8	*PRRT4*	−0.86	6.32 × 10^−4^
ENSG00000147689.16	*FAM83A*	−0.75	2.76 × 10^−3^
ENSG00000121933.17	*ADORA3*	−0.74	4.21 × 10^−2^
ENSG00000100320.22	*RBFOX2*	−0.59	7.27 × 10^−3^
ENSG00000144648.14	*ACKR2*	0.59	3.45 × 10^−2^
ENSG00000165895.17	*ARHGAP42*	0.59	4.09 × 10^−8^
ENSG00000143479.15	*DYRK3*	0.59	6.97 × 10^−9^
ENSG00000183111.11	*ARHGEF37*	0.60	6.90 × 10^−4^
ENSG00000181856.14	*SLC2A4*	0.61	6.72 × 10^−3^
ENSG00000154114.12	*TBCEL*	0.62	2.26 × 10^−11^
ENSG00000188175.9	*HEPACAM2*	0.62	3.50 × 10^−7^
ENSG00000108309.12	*RUNDC3A*	0.63	1.02 × 10^−3^
ENSG00000162543.5	*UBXN10*	0.63	3.89 × 10^−9^
ENSG00000122644.12	*ARL4A*	0.66	3.01 × 10^−9^
ENSG00000152484.13	*USP12*	0.66	7.20 × 10^−11^
ENSG00000102904.14	*TSNAXIP1*	0.67	9.05 × 10^−4^
ENSG00000196407.11	*THEM5*	0.67	2.28 × 10^−2^
ENSG00000134184.12	*GSTM1*	0.69	2.86 × 10^−4^
ENSG00000165061.14	*ZMAT4*	0.72	3.47 × 10^−2^
ENSG00000111339.10	*ART4*	0.75	2.49 × 10^−9^
ENSG00000198892.6	*SHISA4*	0.78	6.50 × 10^−3^
ENSG00000163827.12	*LRRC2*	0.80	9.63 × 10^−11^
ENSG00000188582.8	*PAQR9*	0.80	1.78 × 10^−5^
ENSG00000066468.20	*FGFR2*	0.88	2.91 × 10^−2^
ENSG00000179914.4	*ITLN1*	1.09	7.95 × 10^−7^
ENSG00000176641.10	*RNF152*	1.18	2.88 × 10^−13^
ENSG00000145423.4	*SFRP2*	1.40	2.44 × 10^−13^
ENSG00000188536.12	*HBA2*	1.45	5.37 × 10^−6^
ENSG00000276345.1	*AC004556.1*	1.54	2.16 × 10^−6^
ENSG00000206172.8	*HBA1*	1.68	2.23 × 10^−4^
ENSG00000249992.1	*TMEM158*	1.69	2.08 × 10^−14^
ENSG00000180537.12	*RNF182*	1.78	5.49 × 10^−17^

* Significantly differentially expressed transcripts with a *q*-value < 0.05, an absolute fold-change ≥ 1.5, and expression in at least 50% of samples (TPM > 0.5). Abbreviations: IA = intracranial aneurysm, F-C = fold-change.

## Data Availability

Data is available upon reasonable request to the corresponding author.

## Supplementary Materials

The following are available online at https://www.mdpi.com/article/10.3390/jpm13020266/s1, Table S1: RNA Quality and Sequencing QC Metrics, Table S2: IPA Networks for PAT-Associated Differentially Expressed Genes, Table S3: Significant IPA Disease and Biological Functions for PAT-Associated Differentially Expressed Genes, Figure S1: CIBERSORT Deconvolution, Figure S2: PCA Based on DEG Levels in the Testing Dataset.
