# Supplementary material for: RNA Expression Signatures of Intracranial Aneurysm Growth Trajectory Identified in Circulating Whole Blood
Source: J Pers Med. 2023 Jan 31;13(2):266. doi: 10.3390/jpm13020266 (PMC9967913; doi:10.3390/jpm13020266)
Supplement: Supplementary file 1 [file jpm-13-00266-s001.zip › jpm-2165218-supplementary.pdf]

Supplemental Information for: RNA Expression Signatures of Intracranial Aneurysm Growth Trajectory Identified in Circulating Whole Blood

Kerry E. Poppenberg PhD[1,2], Aichi Chien PhD[4], Briana A. Santo BS[1,3], Ammad A. Baig MD[1,2], Andre Monteiro MD[1,2], Adam A. Dmytriw MD MPH MSc[5], Jan-Karl Burkhardt MD[6], Maxim Mokin MD-PhD[7], Kenneth V. Snyder MD-PhD[1,2], Adnan H. Siddiqui MD-PhD[1,2], Vincent M. Tutino PhD[1,2,3]

[1]Canon Stroke and Vascular Research Center; [2]Department of Neurosurgery, [3]Department of Pathology and Anatomical Sciences, University at Buffalo, Buffalo, NY, USA
[4]Department of Radiology, University of California Los Angeles, Los Angeles, CA, USA
[5]Neuroendovascular Program, Massachusetts General Hospital, Harvard Medical School, Boston, MA, USA
[6]Department of Neurosurgery, University of Pennsylvania, Philadelphia, PA, USA
[7]Department of Neurosurgery, University of South Florida, Tampa, FL, USA

*** Corresponding Author:**
Vincent M. Tutino, PhD
875 Ellicott Street
Canon Stroke and Vascular Research Center
University at Buffalo
Buffalo, NY 14203, USA
E-mail: vincentt@buffalo.edu
Phone: (716) 829-5400

Supplemental Tables

Supplemental Table S1. RNA Quality and Sequencing QC Metrics.*

Sample ID	260/280	RIN	% Assigned	M Assigned	% Aligned
1	2.04	10.00	0.271	15.1	0.96
2	2.00	8.77	0.192	16.0	0.957
3	2.00	8.46	0.228	24.1	0.965
4	2.00	8.46	0.181	20.9	0.963
5	1.85	8.25	0.206	13.7	0.959
6	2.03	9.49	0.237	18.3	0.965
7	1.98	9.49	0.218	17.3	0.965
8	1.98	9.59	0.277	17.6	0.95
9	1.66	7.40	0.202	10.4	0.958
10	1.87	6.70	0.040	8.0	0.98
11	1.93	6.50	0.226	15.0	0.968
12	2.04	9.38	0.240	18.4	0.967
13	2.06	8.90	0.219	19.3	0.968
14	2.01	9.80	0.249	14.5	0.964
15	1.92	7.60	0.253	15.1	0.962
16	1.92	6.00	0.221	14.5	0.951
17	1.95	6.80	0.284	16.3	0.962
18	1.97	9.20	0.277	19.2	0.955
19	2.04	8.90	0.230	15.1	0.964
20	2.02	7.20	0.242	20.2	0.961
21	1.95	6.10	0.093	15.6	0.974
22	1.96	8.90	0.120	18.3	0.972
23	1.96	6.10	0.230	9.3	0.962
24	1.96	7.84	0.224	15.9	0.954
25	2.06	9.00	0.252	17.9	0.962
26	2.03	9.10	0.268	25.2	0.964
27	2.02	9.00	0.266	21.5	0.961
28	1.97	7.90	0.241	17.5	0.966
29	2.04	9.38	0.252	24.1	0.963
30	2.02	9.07	0.246	9.7	0.971
31	2.05	9.07	0.243	11.0	0.973
32	2.04	8.97	0.248	11.6	0.974
33	1.92	8.66	0.265	10.8	0.974
34	2.03	9.07	0.263	11.7	0.974
35	2.07	8.56	0.237	22.9	0.966
36	2.03	8.77	0.255	12.8	0.971
37	2.06	9.28	0.246	10.4	0.971

38	2.06	9.18	0.25	12.3	0.971
39	1.97	8.15	0.235	9.2	0.972
40	2.03	8.87	0.257	12.8	0.968
41	1.97	9.69	0.225	7.3	0.969
42	2.05	9.07	0.270	6.6	0.969
43	2.05	8.56	0.269	14.2	0.972
44	1.99	8.66	0.258	11.3	0.97
45	2.05	8.77	0.259	10.7	0.971
46	2.06	8.46	0.254	10.5	0.97
47	2.04	8.15	0.266	10.5	0.972
48	2.03	8.15	0.264	12.4	0.967
49	1.77	8.15	0.253	10.0	0.969
50	2.04	9.18	0.242	13.7	0.969
51	1.82	8.04	0.281	16.6	0.967
52	2.04	8.04	0.247	14.0	0.971
53	2.06	8.87	0.225	11.9	0.968
54	2.01	8.97	0.255	11.5	0.972
55	2.09	7.63	0.307	12.6	0.966
56	1.98	8.15	0.263	12.4	0.974
57	2.04	8.97	0.270	10.5	0.969
58	1.95	8.56	0.232	14.3	0.967
59	1.05	7.94	0.289	12.6	0.967
60	2.01	7.94	0.230	9.8	0.973
61	2.05	8.66	0.246	11.6	0.965
62	2.05	8.46	0.280	11.6	0.953
63	1.77	8.87	0.278	11.9	0.967
64	2.05	8.56	0.257	10.7	0.97
65	2.06	7.73	0.260	12.1	0.967
66	2.01	8.77	0.216	19.7	0.96

* “Assigned” refers to alignments assigned to genome features, whereas “aligned” refers to aligned reads that overlap genes. Abbreviations: QC=quality control, RIN=RNA integrity number, M=million

Supplemental Table S2. IPA Networks for PAT-Associated Differentially Expressed Genes.*

ID	Molecules in Network	P-Score	# Focus Molecules	Top Diseases and Functions
1	17, 18-dihydroxyeicosapentaenoic acid, ACKR2, Akt, AMPK, C1QL3, DEFA1 (includes others), DYRK3, ERK1/2, FAM83A, FGFR2, FSH, Growth hormone, GSTM1, HBA1/HBA2, Histone h3, IFI27, Immunoglobulin, Insulin, ITLN1, LPL, NDUFC1, NFkB (complex), P38 MAPK, Pka, PRRT4, RAP1GAP, RBFOX2, ribose, RNF152, SFRP2, SLC2A14, SLC2A4, TMIGD3, USP12, ZMAT4	49	19	Dermatological diseases and conditions, nutritional disease, organismal injury and abnormalities
2	ALKBH5, APP, ARHGAP42, ARHGEF37, ARL4A, ARMC12, ART4, ATP1B1, CRK, CYP51A1, DDX39A, DKK2, FLRT3, FZD3, glycosylphosphatidylinositol, GOLGA8K (includes others), HSPA8, Hspg, MAP1A, MPZ, OGT, OTOF, P2RX6, PAQR9, PIWIL4, RNF182, RUNDC3A, SDHD, SHOX2, TBCEL, TGFB1, THOC6, TLL1, TSNAXIP1, VIM	25	11	Cardiovascular system development and function, cellular movement, molecular transport

*Abbreviations: IPA=Ingenuity Pathway Analysis, PAT=predicted aneurysm trajectory

Supplemental Table S3. Significant IPA Disease and Biological Functions for PAT-Associated Differentially Expressed Genes.*

Categories	Diseases or Functions Annotation	*p*-value	Molecules [#]
Cancer, Organismal Injury and Abnormalities	Carcinoma	0.000131	ACKR2, ARHGAP42, ARHGEF37, ARL4A, ART4, DEFA1 (includes others), DYRK3, FAM83A, FGFR2, GOLGA8K (includes others), GSTM1, HBA1/HBA2, HEPACAM2, IFI27, IFI44L, ITLN1, LPL, LRRC2, OTOF, PAQR9, PRRT4, RAP1GAP, RBFOX2, RNF152, RNF182, RUNDC3A, SFRP2, SHISA4, SLC2A4, TBCEL, THEM5, TMEM158, TMIGD3, TSNAXIP1, UBXN10, USP12, ZMAT4 [37]
Cancer, Organismal Injury and Abnormalities	Extracranial solid tumor	0.000512	ACKR2, ARHGAP42, ARHGEF37, ARL4A, ART4, DEFA1 (includes others), DYRK3, FAM83A, FGFR2, GOLGA8K (includes others), GSTM1, HBA1/HBA2, HEPACAM2, IFI27, IFI44L, ITLN1, LPL, LRRC2, OTOF, PAQR9, PRRT4, RAP1GAP, RBFOX2, RNF152, RNF182, RUNDC3A, SFRP2, SHISA4, SLC2A4, TBCEL, THEM5, TMEM158, TMIGD3, TSNAXIP1, UBXN10, USP12, ZMAT4 [37]
Cancer, Organismal Injury and Abnormalities	Extrapancreatic malignant tumor	0.00877	ACKR2, ARHGAP42, ARHGEF37, ARL4A, DEFA1 (includes others), DYRK3, FAM83A, FGFR2, GOLGA8K (includes others), GSTM1, HBA1/HBA2, HEPACAM2, IFI27, IFI44L, ITLN1, LPL, LRRC2, OTOF, PAQR9, PRRT4, RAP1GAP, RBFOX2, RNF182, RUNDC3A, SFRP2, SHISA4, SLC2A4, TBCEL, THEM5, TMEM158, TMIGD3, TSNAXIP1, UBXN10, USP12, ZMAT4 [35]
Cancer, Organismal Injury and Abnormalities	Abdominal carcinoma	0.00312	ACKR2, ARHGAP42, ARHGEF37, ARL4A, DEFA1 (includes others), DYRK3, FAM83A, FGFR2, GOLGA8K (includes others), GSTM1, HBA1/HBA2, HEPACAM2, IFI27, IFI44L, ITLN1, LPL, LRRC2, OTOF, PAQR9, PRRT4, RAP1GAP, RBFOX2, RNF182, RUNDC3A, SFRP2, SHISA4, SLC2A4, TBCEL, TMEM158, TMIGD3, TSNAXIP1, UBXN10, USP12, ZMAT4 [34]
Cancer, Organismal Injury and Abnormalities	Intraabdominal organ tumor	0.00655	ACKR2, ARHGAP42, ARHGEF37, ARL4A, DEFA1 (includes others), DYRK3, FAM83A, FGFR2, GOLGA8K (includes others), GSTM1, HBA1/HBA2, HEPACAM2, IFI27, IFI44L, ITLN1, LPL, LRRC2, OTOF, PAQR9, PRRT4, RAP1GAP, RBFOX2,

			RNF182, RUNDC3A, SFRP2, SHISA4, SLC2A4, TBCEL, TMEM158, TMIGD3, TSNAXIP1, UBXN10, USP12, ZMAT4 [34]
Cancer, Gastrointestinal Disease, Organismal Injury and Abnormalities	Digestive system cancer	0.0175	ACKR2, ARHGAP42, ARHGEF37, DEFA1 (includes others), DYRK3, FAM83A, FGFR2, GOLGA8K (includes others), GSTM1, HBA1/HBA2, HEPACAM2, IFI27, IFI44L, LPL, LRRC2, OTOF, PAQR9, PRRT4, RAP1GAP, RBFOX2, RNF182, RUNDC3A, SFRP2, SHISA4, SLC2A4, TBCEL, TMEM158, TMIGD3, TSNAXIP1, UBXN10, USP12, ZMAT4 [32]
Cancer, Organismal Injury and Abnormalities	Melanoma	0.000895	ACKR2, ARHGAP42, ARHGEF37, ART4, DEFA1 (includes others), DYRK3, FAM83A, FGFR2, GOLGA8K (includes others), GSTM1, HEPACAM2, IFI44L, ITLN1, LPL, LRRC2, OTOF, PRRT4, RAP1GAP, RBFOX2, RNF152, RNF182, RUNDC3A, SHISA4, SLC2A4, TBCEL, TMIGD3, TSNAXIP1, UBXN10, USP12, ZMAT4 [30]
Cancer, Organismal Injury and Abnormalities	Head and neck cancer	0.0227	ARHGAP42, ARHGEF37, ARL4A, DEFA1 (includes others), FAM83A, FGFR2, GOLGA8K (includes others), GSTM1, HEPACAM2, IFI27, IFI44L, ITLN1, LPL, LRRC2, OTOF, PAQR9, PRRT4, RAP1GAP, RBFOX2, RUNDC3A, SFRP2, SHISA4, SLC2A4, TBCEL, THEM5, TMEM158, TMIGD3, TSNAXIP1, USP12, ZMAT4 [30]
Cancer, Dermatological Diseases and Conditions, Organismal Injury and Abnormalities	Skin cancer	0.00201	ACKR2, ARHGAP42, ARHGEF37, ART4, DEFA1 (includes others), DYRK3, FAM83A, FGFR2, GSTM1, HEPACAM2, IFI27, IFI44L, ITLN1, LPL, LRRC2, OTOF, PAQR9, PRRT4, RAP1GAP, RNF152, RNF182, RUNDC3A, SLC2A4, TBCEL, TMIGD3, TSNAXIP1, UBXN10, USP12, ZMAT4 [29]
Cancer, Organismal Injury and Abnormalities	Anogenital cancer	0.00427	ARHGAP42, ARHGEF37, ARL4A, DEFA1 (includes others), DYRK3, FGFR2, GOLGA8K (includes others), GSTM1, HBA1/HBA2, HEPACAM2, IFI27, IFI44L, ITLN1, LPL, LRRC2, OTOF, PAQR9, PRRT4, RAP1GAP, RBFOX2, RUNDC3A, SFRP2, SLC2A4, TMEM158, TMIGD3, TSNAXIP1, USP12, ZMAT4 [28]
Cancer, Organismal Injury and Abnormalities	Malignant genitourinary solid tumor	0.0153	ARHGAP42, ARHGEF37, ARL4A, DEFA1 (includes others), DYRK3, FGFR2, GOLGA8K (includes others), GSTM1, HBA1/HBA2, HEPACAM2, IFI27, IFI44L, ITLN1, LPL, LRRC2, OTOF, PAQR9, PRRT4, RAP1GAP, RBFOX2, RUNDC3A,

			SFRP2, SLC2A4, TMEM158, TMIGD3, TSNAXIP1, USP12, ZMAT4 [28]
Cancer, Dermatological Diseases and Conditions, Organismal Injury and Abnormalities	Cutaneous melanoma	0.0104	ACKR2, ARHGAP42, ARHGEF37, ART4, DEFA1 (includes others), DYRK3, FAM83A, FGFR2, HEPACAM2, IFI44L, ITLN1, LPL, LRRC2, OTOF, PRRT4, RAP1GAP, RNF152, RNF182, RUNDC3A, SLC2A4, TBCEL, TMIGD3, TSNAXIP1, UBXN10, USP12, ZMAT4 [26]
Cancer, Organismal Injury and Abnormalities	Multiple cancers	0.0113	DYRK3, FGFR2, GOLGA8K (includes others), GSTM1, HBA1/HBA2, IFI27, IFI44L, ITLN1, LPL, OTOF, RAP1GAP, RUNDC3A, SFRP2, SLC2A4, TMEM158, TMIGD3, TSNAXIP1, USP12 [18]
Cancer, Organismal Injury and Abnormalities, Respiratory Disease	Lung carcinoma	0.0143	ACKR2, ARHGAP42, FAM83A, FGFR2, GOLGA8K (includes others), GSTM1, IFI27, IFI44L, ITLN1, OTOF, PRRT4, SFRP2, SLC2A4, TMIGD3, TSNAXIP1, USP12, ZMAT4 [17]
Cancer, Organismal Injury and Abnormalities, Reproductive System Disease	Breast or ovarian cancer	0.0197	DYRK3, FGFR2, GSTM1, HBA1/HBA2, IFI27, IFI44L, ITLN1, LPL, OTOF, RAP1GAP, RUNDC3A, SFRP2, SLC2A4, TMEM158, TMIGD3, TSNAXIP1, USP12 [17]
Cancer, Organismal Injury and Abnormalities, Reproductive System Disease	Prostate cancer	0.023	ARHGAP42, ARHGEF37, DEFA1 (includes others), DYRK3, FGFR2, GOLGA8K (includes others), HBA1/HBA2, HEPACAM2, IFI44L, LRRC2, OTOF, PRRT4, SLC2A4, TSNAXIP1, ZMAT4 [15]
Cancer, Neurological Disease, Organismal Injury and Abnormalities	Brain cancer	0.00241	ARHGAP42, ARHGEF37, FAM83A, FGFR2, LPL, OTOF, PRRT4, RAP1GAP, SFRP2, SHISA4, SLC2A4, TBCEL, THEM5, TSNAXIP1 [14]
Cancer, Organismal Injury and Abnormalities, Reproductive System Disease	Breast cancer	0.0201	DYRK3, FGFR2, GSTM1, HBA1/HBA2, IFI44L, LPL, OTOF, RAP1GAP, RUNDC3A, SFRP2, SLC2A4, TMEM158, TSNAXIP1, USP12 [14]
Cancer, Neurological Disease, Organismal Injury and Abnormalities	Gliomatosis cerebri	0.00385	ARHGAP42, ARHGEF37, FAM83A, LPL, OTOF, PRRT4, RAP1GAP, SFRP2, SHISA4, SLC2A4, TBCEL, THEM5, TSNAXIP1 [13]
Infectious Diseases, Organismal Injury and Abnormalities	Viral Infection	0.0199	DEFA1 (includes others), FGFR2, GSTM1, HBA1/HBA2, IFI27, IFI44L, ITLN1, LPL, SLC2A4, THEM5 [10]
Inflammatory Response, Organismal Injury and Abnormalities	Inflammation of organ	0.0152	ACKR2, DEFA1 (includes others), FGFR2, GSTM1, HBA1/HBA2, IFI27, IFI44L, LPL, SLC2A4 [9]

Infectious Diseases, Organismal Injury and Abnormalities	Infection by RNA virus	0.0086	DEFA1 (includes others), GSTM1, HBA1/HBA2, IFI27, IFI44L, LPL, SLC2A4, THEM5 [8]
Infectious Diseases, Organismal Injury and Abnormalities	COVID-19	0.000545	GSTM1, HBA1/HBA2, IFI27, IFI44L, SLC2A4, THEM5 [6]
Dermatological Diseases and Conditions, Inflammatory Disease, Inflammatory Response, Organismal Injury and Abnormalities	Dermatitis	0.000953	ACKR2, DEFA1 (includes others), IFI27, IFI44L, LPL, SLC2A4 [6]
Endocrine System Disorders, Gastrointestinal Disease, Metabolic Disease, Organismal Injury and Abnormalities	Non-insulin-dependent diabetes mellitus	0.00102	HBA1/HBA2, ITLN1, LPL, SLC2A4, USP12, ZMAT4 [6]
Dermatological Diseases and Conditions, Organismal Injury and Abnormalities	Psoriasis	0.00159	ACKR2, DEFA1 (includes others), FGFR2, GSTM1, IFI27, IFI44L [6]
Connective Tissue Development and Function, Tissue Morphology	Quantity of connective tissue	0.0158	DYRK3, FGFR2, HBA1/HBA2, LPL, SLC2A4 [5]
Cellular Development, Connective Tissue Development and Function, Tissue Development	Differentiation of connective tissue cells	0.0227	ARL4A, FGFR2, LPL, SFRP2, SLC2A4 [5]
Organismal Injury and Abnormalities, Renal and Urological Disease	Renal impairment	0.00328	GSTM1, HBA1/HBA2, LPL, SLC2A4 [4]
Organismal Injury and Abnormalities, Renal and Urological Disease	Chronic kidney disease	0.00391	GSTM1, HBA1/HBA2, LPL, SLC2A4 [4]
Cellular Development, Connective Tissue Development and Function, Tissue Development	Differentiation of adipocytes	0.00486	ARL4A, LPL, SFRP2, SLC2A4 [4]
Cancer, Hematological Disease, Immunological	Plasma cell myeloma	0.0127	FGFR2, GOLGA8K (includes others), LPL, USP12 [4]

Disease, Organismal Injury and Abnormalities			
Gastrointestinal Disease, Hepatic System Disease, Metabolic Disease, Organismal Injury and Abnormalities	Hepatic steatosis	0.0134	FGFR2, LPL, SLC2A4, THEM5 [4]
Organismal Injury and Abnormalities	Idiopathic fibrosis	0.0145	DEFA1 (includes others), FGFR2, IFI27, SFRP2 [4]
Cell-To-Cell Signaling and Interaction	Binding of tumor cell lines	0.0173	FGFR2, ITLN1, LPL, RAP1GAP [4]
Immunological Disease, Inflammatory Disease, Organismal Injury and Abnormalities	Systemic lupus erythematosus	0.0202	GSTM1, IFI27, IFI44L, SLC2A4 [4]
Hereditary Disorder, Metabolic Disease, Organismal Injury and Abnormalities	ATAD3A-related type I interferonopathy	0.00000709	IFI27, IFI44L, OTOF [3]
Nutritional Disease, Organismal Injury and Abnormalities	Undernutrition	0.000261	FGFR2, HBA1/HBA2, SLC2A4 [3]
Cell-To-Cell Signaling and Interaction	Binding of colorectal cancer cell lines	0.000313	FGFR2, ITLN1, RAP1GAP [3]
Cancer, Dermatological Diseases and Conditions, Organismal Injury and Abnormalities	Development of skin tumor	0.000422	ACKR2, FGFR2, GSTM1 [3]
Cellular Movement, Hematological System Development and Function, Immune Cell Trafficking, Inflammatory Response, Lymphoid Tissue Structure and Development	Chemotaxis of lymphocytes	0.00199	ACKR2, DEFA1 (includes others), RAP1GAP [3]
Cell Death and Survival	Cell survival of tumor cell lines	0.00249	DYRK3, FGFR2, GSTM1 [3]
Infectious Diseases, Organismal Injury and Abnormalities	Severe COVID-19	0.0037	IFI27, IFI44L, SLC2A4 [3]
Organismal Injury and Abnormalities, Renal	Chronic renal impairment	0.00722	GSTM1, LPL, SLC2A4 [3]

and Urological Disease			
Cardiovascular Disease, Organismal Injury and Abnormalities	Ischemia of heart	0.00845	GSTM1, LPL, SLC2A4 [3]
Cardiovascular Disease, Organismal Injury and Abnormalities	Peripheral arterial disease	0.0113	HBA1/HBA2, LPL, SLC2A4 [3]
Cancer, Organismal Injury and Abnormalities	Advanced adenocarcinoma	0.0154	FGFR2, ITLN1, SLC2A4 [3]
Dermatological Diseases and Conditions, Immunological Disease, Inflammatory Disease, Inflammatory Response, Organismal Injury and Abnormalities	Atopic dermatitis	0.0158	DEFA1 (includes others), IFI27, LPL [3]
Organismal Injury and Abnormalities, Renal and Urological Disease	Failure of kidney	0.0175	GSTM1, HBA1/HBA2, SLC2A4 [3]
Hematological Disease, Metabolic Disease, Organismal Injury and Abnormalities	Dysglycemia	0.0187	FGFR2, LPL, SLC2A4 [3]
Carbohydrate Metabolism, Molecular Transport, Small Molecule Biochemistry	Uptake of D-glucose	0.0188	FGFR2, LPL, SLC2A4 [3]
Cardiovascular Disease, Organismal Injury and Abnormalities, Skeletal and Muscular Disorders	Myocardial infarction	0.0203	GSTM1, LPL, SLC2A4 [3]

*Abbreviations: IPA=Ingenuity Pathway Analysis, PAT=Predicted aneurysm trajectory

Supplemental Figures

Supplemental Figure S1

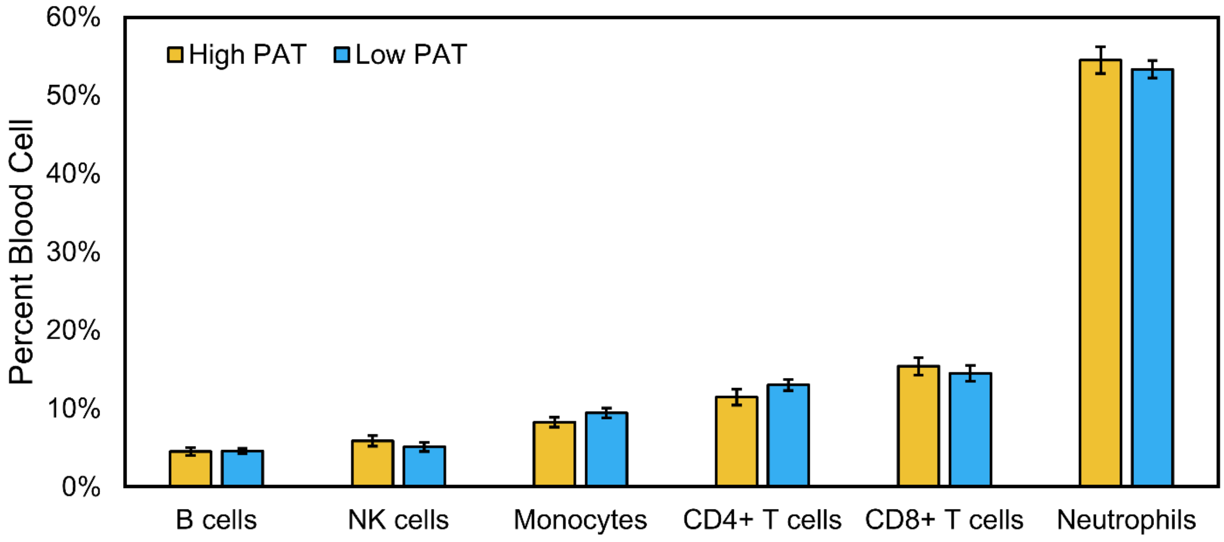

Supplemental Figure S1. CIBERSORT Deconvolution. In both "stable" (low PAT) and "growing" (high PAT) IA groups, neutrophils comprised the majority of the signal, followed by CD8 T cells, CD4 T cells, monocytes, natural killer cells, and B cells. There were no statistically significant differences in percent blood cells between the two groups. (Abbreviations: NK=natural killer)

Supplemental Figure S2

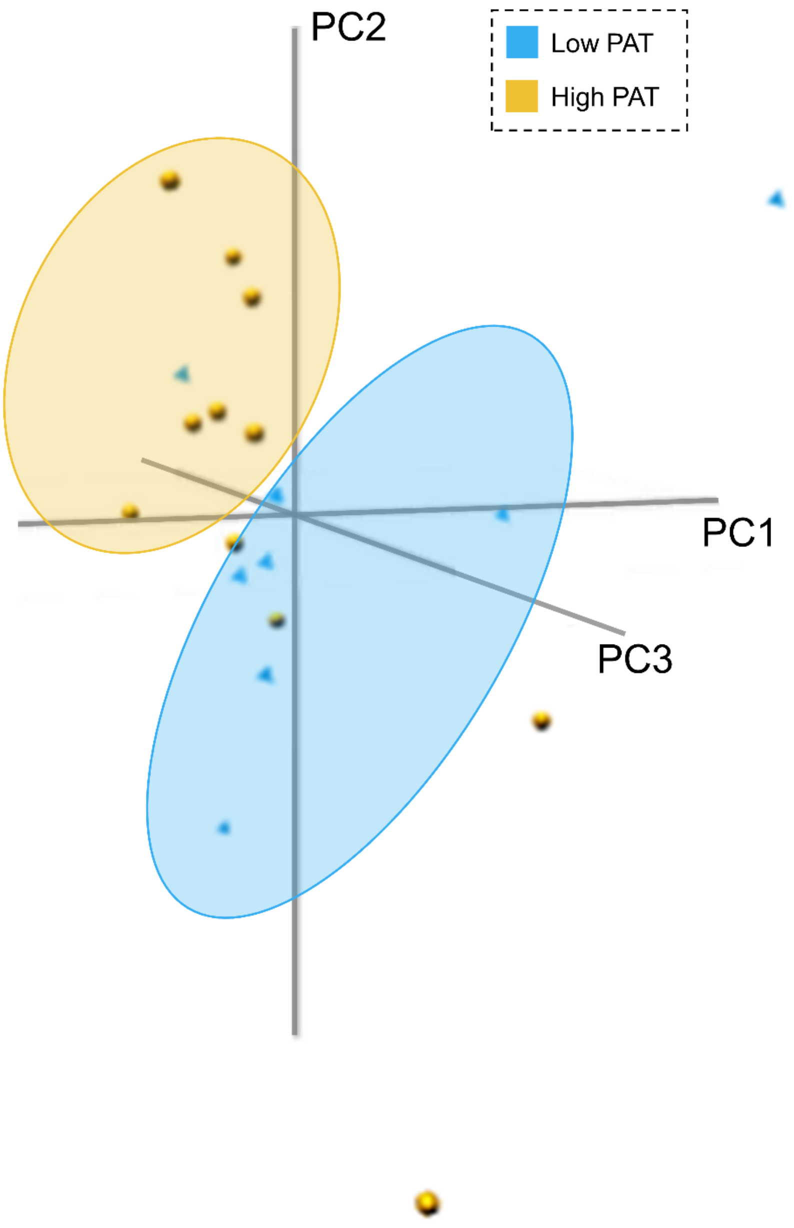

Supplemental Figure S2. PCA Based on DEG Levels in the Testing Dataset. The three-dimensional principal component analysis using expression of PAT-associated DEGs in testing dataset reflects modest separation between low-risk ("stable") and high-risk ("growing") IAs. (Abbreviations: PC=principal component, PAT=predicted aneurysm trajectory, DEG=differentially expressed gene)
